# A loss of heterozygosity, a loss in competition? The effects of inbreeding, pre‐ and postnatal conditions on nestling development

**DOI:** 10.1002/ece3.2496

**Published:** 2016-10-11

**Authors:** Raïssa A. de Boer, Marcel Eens, Wendt Müller

**Affiliations:** ^1^ Department of Biology Behavioural Ecology and Ecophysiology Group – University of Antwerp Wilrijk Belgium

**Keywords:** canary, hatching asynchrony, inbreeding–environment interaction, maternal effects, songbird

## Abstract

The early developmental trajectory is affected by genetic and environmental factors that co‐depend and interact often in a complex way. In order to distinguish their respective roles, we used canaries (*Serinus canaria*) of different genetic backgrounds (inbred and outbred birds). An artificial size hierarchy was created to provoke within‐nest competition, manipulating postnatal conditions. To this end, inbred birds were weight‐matched with outbred birds into duos, and each nest contained one duo of size‐advantaged, and one duo of size‐disadvantaged inbred and outbred nestlings. Prenatal (maternal) effects were taken into account also, enabling us to study the separate as well as the interactive effects of inbreeding, pre‐ and postnatal conditions on nestling development. We find that postnatal conditions were the most important determinant of early growth, with size‐advantaged nestlings growing faster and obtaining larger size/body mass at fledging in comparison with size‐disadvantaged nestlings. Prenatal conditions were important too, with birds that hatched from eggs that were laid late in the laying order obtaining a larger size at fledging than those hatched from early laid eggs. Inbreeding inhibited growth, but surprisingly this did not depend on (dis)advantageous pre‐ or postnatal conditions. Our findings imply that inbred individuals lose when they are in direct competition with same‐sized outbred individuals regardless of the rearing conditions, and we thus propose that reduced competitiveness is one of the driving forces of inbreeding depression.

## 1 Introduction

The early developmental trajectory is an important determinant of key life history traits such as reproduction, aging, and life span (Gilbert, 2005; Metcalfe & Monaghan, 2001; Monaghan, 2008; Mousseau & Fox, 1998). Comprehending the causes of among‐individual variation in early development can therefore enhance our understanding of various ecological and evolutionary processes. The foremost source of variation in early development is the genotype, which sets the developmental limits for the individual (Metcalfe & Monaghan, 2001) and determines how an individual responds to environmental conditions (Gilbert, 2005). The environmental conditions experienced during early life constitute the second important source of variation in early development and can refer to pre‐ or postnatal conditions according to the period during which they act (Monaghan, 2008).

The early postnatal environment in birds, at least in altricial species that raise more than one offspring at a time, is above all shaped by siblings and the hierarchy among them (Forbes, 2010; Mainwaring, Dickens, & Hartley, 2010). In such species, nestlings are dependent on their parents for food, which is provided in response to begging behavior. However, because larger nestlings may reach closer to their parents while begging, push aside smaller siblings, and position themselves in favored feeding positions of the parents, large nestlings will most often be advantaged in food acquisition in comparison with smaller nestlings (e.g., Oddie, 2000; Royle, Hartley, & Parker, 2002). From a proximate point of view, within‐nest size differences arise in particular if females start to incubate the eggs before having laid the last egg, which will cause the eggs to hatch asynchronously (Magrath, 1989; Mock & Forbes, 1995). Thus, hatching asynchrony will lead to differences in age, and consequently size, with later‐hatched nestlings having a disadvantage in sibling competition in comparison with older siblings.

The prenatal environment is another important element of the early life conditions, which is in birds to a large extent shaped by mothers via differential allocation in eggs. This can relate to differences in egg size along the laying order, which can affect size at hatching and/or nutrient availability (Christians, 2002; Royle, Surai, McCartney, & Speake, 1999; Williams, 1994). Moreover, the contents of the egg can also differ along the laying order, for example, varying levels of hormones (Gil, Graves, Hazon, & Wells, 1999; Groothuis & Schwabl, 2002; Royle, Surai, & Hartley, 2001; Schwabl, 1993) and antioxidants (Blount et al., 2002; Royle, Surai, & Hartley, 2003; Royle et al., 1999, 2001). These maternally derived egg components can substantially affect further development of the offspring (referred to as maternal effects) and are thought to navigate the developmental trajectory according to the expected environmental conditions (Groothuis, Müller, von Engelhardt, Carere, & Eising, 2005; Muller & Groothuis, 2013). Thus, the extent to which maternal effects can (adaptively) influence development is often dependent on the posthatching environmental conditions experienced by the offspring (Marshall & Uller, 2007). For example, the testosterone content of the yolk increases with the laying order of the egg, which can stimulate faster growth and in this way prepare late‐hatched nestlings for their size‐disadvantaged position in the sibling hierarchy (Eising & Eikenaar, 2001; Groothuis et al., 2005; Müller, Boonen, Groothuis, & Eens, 2010; Muller & Groothuis, 2013; Schwabl, 1993, 1996).

Such maternal aggravation or alleviation of sibling competition via maternal effects exemplifies that pre‐ and postnatal environmental conditions are co‐dependent. However, not only pre‐ and postnatal environmental conditions are likely affecting offspring development in interplay, but an individual's response will also depend on genetic aspects of its condition. For example, a poor genetic makeup as a result from mating between related individuals (=inbreeding) can enhance the negative effects of adverse environmental conditions on the individual (Fox & Reed, 2011). Previously, it was shown that inbreeding depression on early growth was especially noticeable in small, late‐hatched nestlings, indicating that negative effects of hatching asynchrony interacted with inbreeding (de Boer, Eens, Fransen, & Müller, 2015). It is, however, unknown if this inbreeding–environment interaction is caused by differences in competitive ability, maternal effects, or even their interaction. An integrated experimental approach is required to obtain a better understanding of the relative importance of each of these factors.

Here, we investigated how the effects of genetic condition on early development are linked with environmental conditions experienced during the pre‐ and/or postnatal period in canaries (*Serinus canaria)*. To this end, we mated full‐siblings (=inbred group) and unrelated individuals (=outbred group), to create two cohorts that differ in genetic condition. Inbred and outbred nestlings were weight‐matched and reared together in a foster nest, either in a “senior” (age/size advantaged) or in a “junior” (age/size disadvantaged) position. The junior nestlings are thus in an unfavorable condition in comparison with the senior nestlings, mimicking the natural sibling hierarchy as induced by hatching asynchrony, and thus manipulating an important aspect of postnatal conditions. These weight‐matched junior and senior duos were additionally matched for laying position, because as mentioned above, maternally allocated egg contents (e.g., hormones) may vary along the laying order, constituting an important aspect of prenatal conditions. We grouped first or second laid eggs as “A‐eggs,” and all later laid eggs as “B‐eggs,” because the first two eggs typically have the lowest testosterone content, while later laid eggs contain higher amounts (Schwabl, 1996; Vergauwen, Goerlich, Groothuis, Eens, & Müller, 2012). Finally, in order to investigate how maternal effects may modulate effects of hatching asynchrony, we created match and mismatch conditions. That is, nestlings hatched from A‐eggs were reared in a senior position (=matched pre‐ and postnatal conditions) or in a junior position (=mismatched pre‐ and postnatal conditions), and vice versa for nestlings hatched from B‐eggs.

The above‐mentioned experimental design enabled us to assess the relative importance of each factor and their potentially complex interplay on growth rate and size at fledging. We expect that junior nestlings are lagging in growth in comparison with senior nestlings, because the initial size disadvantage of junior nestlings constrains their access to food. Furthermore, we expect that competition may be mitigated by maternal effects, which should thus be particularly important for nestlings reared in a junior position. Last, we expect that heavy competition enhances inbreeding depression and that early growth should therefore be most inhibited in inbred nestlings that were reared in a junior position.

## 2 Material and Methods

### 2.1 Study species and experimental setup

One‐year‐old canaries, originating from an outbred population kept at the University of Antwerp, were used for breeding. The canaries were stimulated into reproductive state by setting the lights at a 14‐hr light, 10‐hr dark regime (starting March 2014). After 5 weeks with this light regime, the experiment commenced. Birds were kept in standard cages (50 × 64 × 40 cm^3^, GEHU cages, the Netherlands), equipped with two perches, shell sand, a nest‐cup, nesting material, and constant access to seeds (Van Camp, Belgium) and water. After breeding pairs had finished constructing a nest, it was checked daily for eggs. Eggs were weighed and marked according to order of laying with a nontoxic marker. Fourteen days after the first egg was laid (=minimal incubation period), nests were checked daily for nestlings that had hatched. Birds were given unlimited access to egg food (Van Camp, Belgium), supplemented with 1 tablespoon/kg Orlux hand mix (Versele‐Laga) and freshly germinated seeds after the first nestling hatched. All nestlings were marked for individual recognition at hatching with a nontoxic colored marker (Artline 70N), until a numbered metal ring could be fitted on its leg. It was also noted from which egg it had hatched, and on what date it hatched. Nestlings were weighed daily in the morning until 15 days after hatching. A sample of blood was taken at fledging (±25 days after hatching) in order to determine sex with the use of PCR. Additionally, birds were weighed at fledging and tarsus length was measured with calipers.

The focal birds used in this study originated from 40 full‐sibling breeding pairs (=94 inbred birds) and 43 breeding pairs in which the partners were unrelated (=94 outbred birds). All birds were cross‐fostered within 2 days after hatching. Birds were cross‐fostered in duos into a foster nest (N = 54). In 32 of these nests, nestlings were reared by unrelated foster parents, and in 22 by full‐sibling foster parents, which was taken into account in the statistical analyses. A duo consisted of an inbred and outbred bird that were matched according to the egg they had originated from (first or second egg= “A‐egg,” all later laid eggs=“B‐eggs”). Additionally, they were matched for weight at time of cross‐fostering (<0.2 g difference). Then, two sets of duos were combined, so that each experimental nest contained four nestlings, a typical brood size for canaries (Estramil, Eens, & Müller, 2013). Two sets of duos were combined based on age: each experimental nest contained an older “senior” duo, and a younger “junior” duo. The difference between seniors and juniors was 2 days in age, which corresponds to the first (hatched day *i*) and third (hatched day *i *+* *2) position in the size hierarchy induced by natural hatching asynchrony (de Boer et al., 2015). If it was not possible to combine nestlings in this way, we combined nestlings that differed at least 0.5 g in weight at time of cross‐fostering which corresponds to at least 1 day difference in age (de Boer et al., 2015). Thus, four experimental treatments were created in both inbred as outbred birds: senior nestlings hatched from A‐eggs (*N*_inbred_ = 25, *N*_outbred_ = 25), senior nestlings hatched from B‐eggs (*N*_inbred_ = 19, *N*_outbred_ = 19), junior nestlings hatched from A‐eggs (*N*_inbred_ = 17, *N*_outbred_ = 17), and junior nestlings hatched from B‐eggs (*N*_inbred_ = 33, *N*_outbred_ = 33). In this way, prenatal effects (hatched from A‐ or B‐eggs) could be separated from postnatal effects (reared in junior or senior position in the within‐nest hierarchy).

### 2.2 Statistical analyses

The growth data were analyzed with nonlinear mixed effects modeling, with the use of the “nlme” package (Pinheiro, Bates, Debroy, Sarkar, & Team, 2016) in R software (R Core Development Team, 2014). To model the growth, we applied a logistic growth curve in the form of: $W_{t}=A/1+e^{\left(K(1-t)\right)}$, with *W*_*t*_ = weight at time *t* (*t* = number of days after hatching), *A* = asymptotic mass, *I* = inflection point, and *K* = growth constant (for more details, see Sofaer, Chapman, Sillett, & Ghalambor, 2013). We included bird identity as a random effect, to correct for the repeated measurements in the data. Further, the nest the birds were reared in was included as a random effect, to correct for the lack of independence due to, among other things, shared parental care. We also included a random effect for the nest of origin, in order to correct for nonindependence of siblings. The model was the best fit if random effects were included for all growth parameters (*A, I,* and *K*). The parameters were allowed to not correlate with each other (i.e., an early inflection point did not have to be associated with a high asymptotic mass).

The fixed effects for the growth parameters were: sex, foster parents (full‐sibling or unrelated), prenatal conditions (=hatched from A or B‐egg), postnatal conditions (=junior or senior), and inbreeding status (=inbred or outbred). In order to test whether the effects of inbreeding were dependent on pre‐ and/or postnatal conditions experienced, we included interactions (inbreeding status × prenatal conditions, inbreeding status × postnatal conditions, prenatal conditions × postnatal conditions, inbreeding status × prenatal conditions × postnatal conditions). Lastly, it was tested whether the effects of inbreeding, prenatal conditions, and postnatal conditions were sex specific by including two‐way interactions between sex and each of these factors. Significance values of the fixed effects were obtained with stepwise backward elimination using log‐likelihood ratio (LR) tests, starting with the highest interaction.

Linear mixed models were used to analyze differences at fledging (=25 days after hatching) in tarsus length and weight. The same random effects, fixed effects, and interactions between fixed effects that were used for the analysis of growth (see above) were used for these analyses. We obtained significance values via stepwise backward elimination with the use of the lmerTest package (Kuznetsova, Brockhoff, & Christensen, 2015).

A binomial generalized linear model was used to analyze differences in the number of nestlings that survived until day 15 according to prenatal condition, postnatal condition, inbreeding, sex, and foster parents. We included the three‐way interaction between inbreeding status, prenatal conditions, and postnatal conditions, and all possible two‐way interactions to test whether differences in survival between inbred and outbred nestlings depended on environmental conditions and whether there were sex‐specific effects. Chi‐squared tests were used to obtain significance values. All results are presented as mean ± *SE*.

## 3 Results

The results of the statistical analyses of the effects of inbreeding, and pre‐ and postnatal conditions on different parameters of growth are summarized in Table 1.

**Table 1:** The results of the stepwise regression for growth (growth constant = *K*, inflection point = *I*, asymptotic mass = *A*), for the effects of pre‐ and postnatal conditions in relation to inbreeding status. Significant results are noted with a star (*)

Growth parameter	Term	LR	*p*‐ value
*K*	Inbreeding status: prenatal conditions: postnatal conditions	0.03	.85
*I*	Inbreeding status: prenatal conditions: postnatal conditions	2.07	.15
*A*	Inbreeding status: prenatal conditions: postnatal conditions	1.67	.20
*K*	Inbreeding status: prenatal conditions	0.00	.98
*I*	Inbreeding status: prenatal conditions	0.59	.44
*A*	Inbreeding status: prenatal conditions	1.00	.32
*K*	Inbreeding status: postnatal conditions	0.70	.40
*I*	Inbreeding status: postnatal conditions	0.75	.39
*A*	Inbreeding status: postnatal conditions	0.05	.83
*K*	Prenatal conditions: postnatal conditions	3.33	.07
*I*	Prenatal conditions: postnatal conditions	0.34	.56
*A*	Prenatal conditions: postnatal conditions	0.25	.62
*K*	Inbreeding status: sex	1.48	.22
*I*	Inbreeding status: sex	0.18	.67
*A*	Inbreeding status: sex	0.12	.73
*K*	Prenatal conditions: sex	0.08	.78
*I*	Prenatal conditions: sex	1.59	.21
*A*	Prenatal conditions: sex	4.71	.03*
*K*	Postnatal conditions: sex	0.62	.43
*I*	Postnatal conditions: sex	0.05	.82
*A*	Postnatal conditions: sex	0.02	.89
*K*	Foster parents	0.06	.80
*I*	Foster parents	0.35	.55
*A*	Foster parents	0.51	.47
*K*	Prenatal conditions	0.15	.70
*I*	Prenatal conditions	0.01	.94
*A*	Prenatal conditions	In interaction	
*K*	Postnatal conditions	15.05	<.001*
*I*	Postnatal conditions	7.68	.0056*
*A*	Postnatal conditions	13.84	<.001*
*K*	Inbreeding status	9.08	.0026*
*I*	Inbreeding status	1.84	.18
*A*	Inbreeding status	0.61	.43
*K*	Sex	0.29	.59
*I*	Sex	2.32	.13
*A*	Sex	In interaction	

### 3.1 Postnatal conditions

Postnatal conditions had large effects on the growth trajectory of canary nestlings. Birds that were reared in a senior position in the nest had larger growth constants (junior: 0.29 ± 0.005, senior: 0.32 ± 0.006), earlier inflection points (junior: 8.4 ± 0.2 days, senior: 7.7 ± 0.2 days), and larger asymptotic masses (junior: 17.1 ± 0.4 g, senior: 18.6 ± 0.5 g).

Tarsus length (*F*_1,137.13_ = 111.12, *p*‐value =.0011) and weight at fledging (*F*_1,139.45_ = 5.16, *p*‐value =.025) were significantly affected by postnatal conditions. Birds reared in junior positions in the nest were smaller (N* *=* *80, tarsus length: 18.12 ± 0.08 mm, weight: 18.73 ± 0.2 g) than those reared in senior positions (N* *=* *85, tarsus length: 18.46 ± 0.07 mm, weight: 19.39 ± 0.2 g).

### 3.2 Prenatal conditions

**Figure 1:**
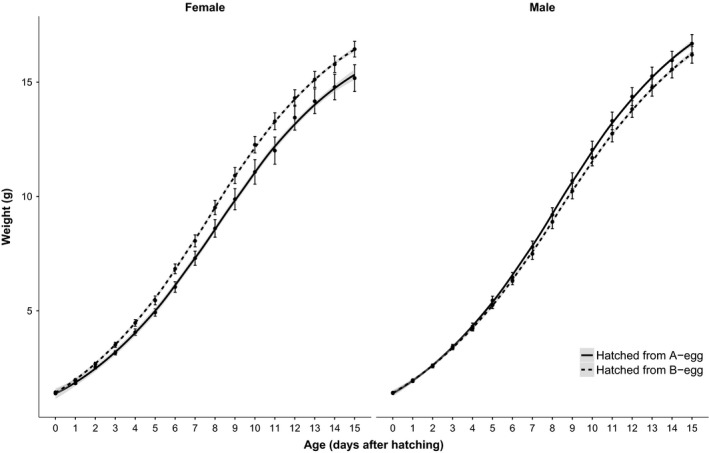
Growth rate of male and female canary nestlings, hatched from a first or second laid egg (A‐egg) or from later laid eggs (B‐egg)

There were sex‐specific effects of prenatal conditions (Table 1). Prenatal effects on growth were more prominent in females than in males (Figure 1): females hatched from B‐eggs obtained larger asymptotic masses in comparison with females hatched from A‐eggs. In males, on the other hand, there was little difference in growth according to prenatal conditions. The effects of prenatal conditions were not dependent on postnatal conditions (Table 1).

Further, prenatal conditions affected tarsus length (*F*_1,125.82_ = 3.98, *p*‐value =.048), but not weight (*F*_1,122.56_ = 2.71, *p*‐value =.10) at fledging. Birds hatched from A‐eggs (N* *=* *74, 18.25 ± 0.09 mm) had smaller tarsus lengths than birds hatched from B‐eggs (N* *=* *91, 18.33 ± 0.07 mm). These effects were not sex specific (tarsus length: *F*_1,141.02_ = 0.06, *p *=* *.81, weight: *F*_1,147.16_ = 2.30, *p*‐value =.13).

### 3.3 Inbreeding

**Figure 2:**
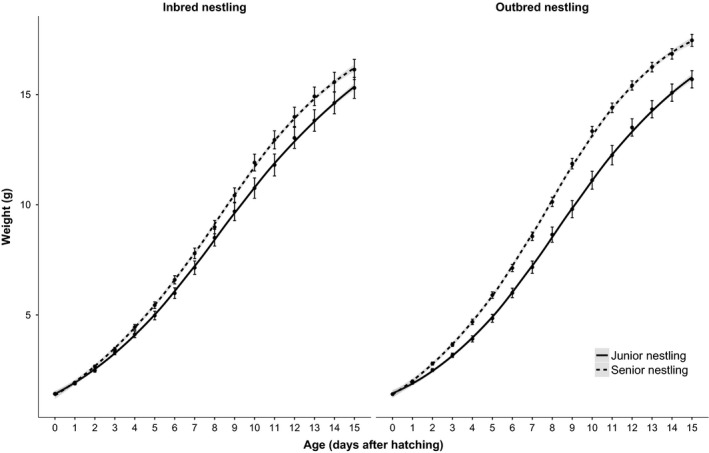
Growth rate of inbred and outbred canary nestlings, reared in a junior (age/size disadvantaged) or senior (age/size advantaged) position in the sibling hierarchy

Inbreeding status significantly affected growth. Inbred nestlings had smaller growth constants (inbred: 0.29 ± 0.006, outbred: 0.31 ± 0.006) in comparison with outbred nestlings, but there were no differences in the inflection point or asymptotic mass according to inbreeding status (Table 1). The effects of inbreeding did not interact with postnatal conditions on any of the growth parameters; the effects of inbreeding were not dependent on size (dis)advantages in the nest (Figure 2).

**Figure 3:**
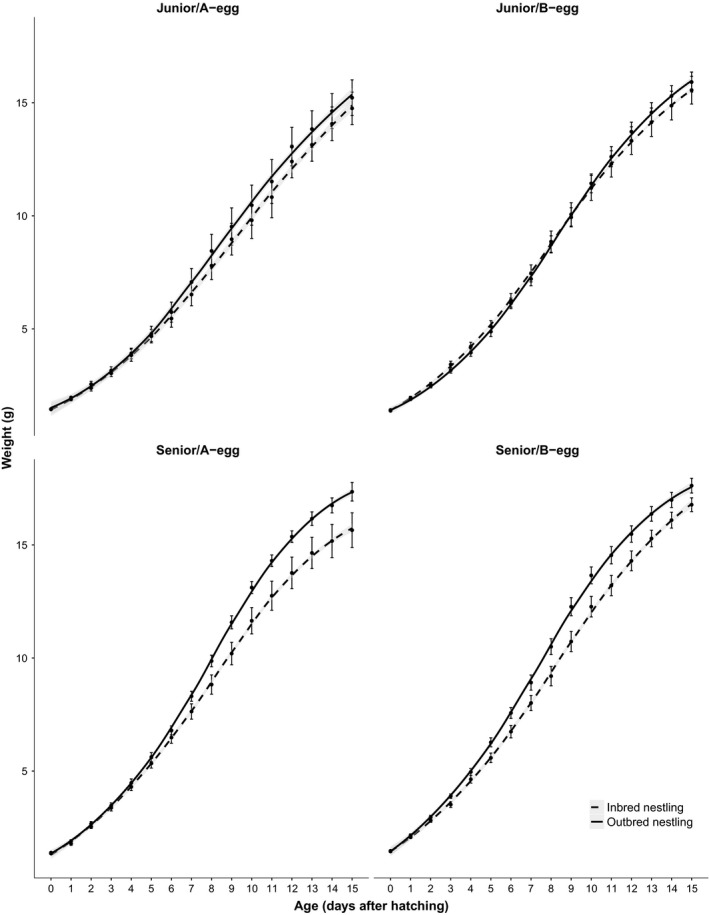
Growth rate of inbred and outbred canary nestlings under different combinations of pre‐ and postnatal conditions: Hatched from a first or second laid egg (A‐egg) or from later laid eggs (B‐egg), reared in a junior (age/size disadvantaged) or in a senior (age/size advantaged) position in the sibling hierarchy

There were also no significant interactions between inbreeding status and prenatal conditions (Table 1). Further, there was no significant three‐way interaction between inbreeding status, and pre‐ and postnatal conditions on any of the growth parameters. Indeed, as visualized in Figure 3, outbred nestlings outgrew inbred nestlings under all conditions, although this was most noticeable in senior nestlings that hatched from A‐eggs.

Inbreeding did not affect weight/size at fledging (tarsus length: *F*_1,41.05_ = 0.18, *p*‐value =.67, weight: *F*_1,46.27_ = 0.70, *p*‐value =.41), neither in interaction with postnatal conditions (tarsus length: *F*_1,110.80_ = 1.05, *p*‐value =.31, weight: *F*_1,113.55_ = 0.34, *p*‐value =.56) nor prenatal conditions (tarsus length: *F*_1,94.23_ = 0.33, *p*‐value =.57, weight: *F*_1,87.35_ = 1.19, *p*‐value =.28).

Independent of any factors, sex affected weight (*F*_1,155.85_ = 9.78, *p*‐value =.0021) and tarsus length at fledging (*F*_1,152.07_ = 4.34, *p*‐value =.039). Males were larger (N* *=* *87, tarsus length: 18.37 ± 0.08 mm, weight: 19.40 ± 0.2 g) than females (N* *=* *78, tarsus length: 18.21 ± 0.07 mm, weight: 18.69 ± 0.2 g).

### 3.4 Survival

Postnatal conditions strongly affected survival until 15 days after hatching (χ^2^(1) = 12.18, *p *=* *.0005). Only one bird that was reared in the senior position did not survive, whereas 14% ± 4% of junior birds did not survive. There was no significant difference in survival according to any of the other factors (all *p*‐values >.08).

In none of the above analyses, was there a significant effect of the foster parents (full sibling or unrelated parents) (all *p*‐values >.4).

## 4 Discussion

We compared the early developmental trajectories of inbred birds with outbred birds under distinct competitive conditions, while accounting for maternal effects. Our unique experimental design enabled us to establish the relative importance of inbreeding, pre‐ and postnatal conditions, and their potential interactions, in a way that most likely would not be achievable in wild populations.

### 4.1 Postnatal conditions

We show that manipulation of the within‐nest size hierarchy, an important aspect of the postnatal conditions which is typically induced by hatching asynchrony, had profound effects on the early development of the birds. As expected, nestlings that were placed in a senior (size advantaged) position grew faster than those placed in a junior (size disadvantaged) position. In addition, senior nestlings were heavier and structurally larger at fledging, and their survival was higher than was found for junior nestlings. These findings imply that we successfully mimicked hatching asynchrony, which generally handicaps the smaller nestlings (Forbes, 2010; Mainwaring et al., 2010). The latter is among other things caused by inhibited food acquisition due to the disadvantage in sibling competition (Viñuela, 1999) and/or differential parental food allocation (Avilés, Parejo, & Rodríguez, 2011; Cotton, Wright, & Kacelnik, 1999; Kilner, 2002). Thus, size asymmetries can even cause differences in nestling growth and survival when parents have ad libitum access to food (see also de Boer et al., 2015), but obviously such effects may become even more pronounced when there is limited access to food. Size (dis)advantages can, thus, determine under which selection pressures, nestlings are reared, which can, similar to our findings, directly affect growth (de Boer et al., 2015; Mainwaring et al., 2010) and survival (Forbes, 2010). Furthermore, there may be physiological effects of food restriction (Giordano, Costantini, & Tschirren, 2015), and there may also be long‐term consequences, for example, on fitness (Mainwaring, Blount, & Hartley, 2012) and personality traits (Mainwaring & Hartley, 2013; Rokka, Pihlaja, Siitari, & Soulsbury, 2014), which we did not analyze here.

### 4.2 Prenatal conditions

We expected that maternal effects would mitigate the effects of sibling hierarchy and that thus specifically size‐disadvantaged junior nestlings (independent of sex) would benefit. But, we did not find that the effects of prenatal conditions interacted with postnatal conditions. Regardless of whether or not the position in the size hierarchy matched the laying order of the egg, there were differences in growth among female nestlings and an overall size difference at fledging according to which egg they hatched from. Birds that hatched from B‐eggs were larger at fledging than those hatched from A‐eggs. We did not measure egg contents in this study, but late laid eggs presumably contained, among other things, less antioxidants (Blount et al., 2002; Royle et al., 1999, 2001, 2003), and less antibodies (Blount et al., 2002) than early laid eggs. It therefore seems contradictory that birds that hatched from late laid eggs benefitted, because birds that hatched from early laid eggs presumably hatched from qualitatively better eggs. The most likely explanation for this finding is a differential allocation of hormones, with late laid eggs containing relatively more androgens, which has consistently been found in captive canaries also under ad libitum feeding conditions (Schwabl, 1993, 1996; Vergauwen et al., 2012). Apparently, this can significantly stimulate growth, and even overrule the effects of the other egg compounds (Eising & Eikenaar, 2001; Groothuis et al., 2005). However, our findings are not congruent with the existing theory on the adaptive significance of maternal effects (Groothuis et al., 2005; Marshall & Uller, 2007; Muller & Groothuis, 2013) as nestlings benefitted independent of the postnatal conditions they encountered. A possible explanation is that nestlings experienced competition with a size‐matched individual in both senior and junior positions, which may have negated the context dependence of maternal effects in our experimental setup.

The prenatal effects were more pronounced and in particular visible at earlier developmental stages among female nestlings. Such sex‐specific effects have been shown before (Müller et al., 2005) and are thought to relate to higher benefits for the smaller sex that is more competitive disadvantaged, here females (Oddie, 2000).

### 4.3 Inbreeding

Inbreeding was an important predictor of early growth; outbred nestlings grew faster in comparison with inbred nestlings. Such negative effects of inbreeding have been described before. In birds specifically, inbreeding has been shown to affect hatching success (de Boer et al., 2015; Spottiswoode & Møller, 2004), growth (de Boer et al., 2015; Bolund, Martin, Kempenaers, & Forstmeier, 2010), and traits in adulthood, such as sexual ornamentation (de Boer et al., 2016; Bolund et al., 2010; Ferrer, García‐Navas, Bueno‐Enciso, Sanz, & Ortego, 2015) and reproductive success (Seddon, Amos, & Mulder, 2004).

However, it was expected that the effects of inbreeding would be enhanced under more stressful conditions (Armbruster & Reed, 2005; Fox & Reed, 2011), and, more specifically, by increased levels of competition (de Boer et al., 2015; Carr & Dudash, 1995; Cheptou, Lepart, & Escarré, 2001; Gallardo & Neira, 2005; Meagher, Penn, & Potts, 2000; Rowe & Beebee, 2004; Valtonen, Roff, & Rantala, 2014). Indeed, it was found in a previous study that the effects of inbreeding were context dependent, being enhanced among last‐hatching nestlings, and it was hypothesized that this inbreeding–environment interaction was caused by sibling competition (de Boer et al., 2015). Therefore, we predicted that the effects of inbreeding should be most noticeable under more intense competition that is in the size‐disadvantaged nestlings.

In contrast, the differences in growth between inbred and outbred nestlings were not affected by the position in the size hierarchy and thus degree of competition. However, there is one major difference in the experimental design between this study and the previous study that may explain this discrepancy. In the previous study (de Boer et al., 2015), broods of inbred nestlings were compared with broods of outbred nestlings, thus in a between‐nest comparison. Whereas here inbred birds were placed in direct competition with weight‐matched outbred birds. Perhaps, small (physiological) differences between inbred and outbred birds were enhanced under the pressure of direct competition, which could be particularly pronounced as competition between equally sized individuals is thought to be most intense (Gilby, Mainwaring, & Griffith, 2011; Merkling et al., 2013; Osorno & Drummond, 1995).

These findings imply that inbred birds are disadvantaged in competition, because inbred individuals obtained slower growth compared to equally sized outbred individuals, regardless of the rearing conditions. This could imply that the loss in competition depends more so on the type of competitor, than on how strong the level of competition is. This is comparable to findings in plants; the negative effects of inbreeding are most noticeable when inbred plants are competing directly with outbred plants (Cheptou et al., 2001; Koelewijn, 2004). Moreover, Cheptou et al. (2001) found that the type of competitor (inbred or outbred plant) was even more important for inbreeding depression than the number of competitors. If we can indeed extrapolate these findings in plants to our findings in birds, and inbred birds lose when they compete with outbred birds regardless of (dis)advantageous conditions, this would be an important factor to consider in the future (vertebrate) animal studies. Unfortunately, we are unaware of what exactly causes a disadvantage in competition, but this could relate to energetic, cognitive, or physiological aspects.

Under natural conditions, inbred nestlings will not compete with outbred nestlings within a brood. However, competition is a key selective pressure throughout an individual's life span, and although inbred birds eventually obtained the same size as outbred birds, if there is some intrinsic effect of inbreeding that causes a bad performance in competition, this can certainly be of importance later in life. In the future, we aim to explore further whether the restrictions in competitiveness are long‐lasting, and if/how the differences in early development are reflected in the adult phenotype.
